# Glucosyl anthranilate

**DOI:** 10.1107/S1600536809040975

**Published:** 2009-10-13

**Authors:** Haoyan Liu, Ailan Zou, Hao Zhang, Xiaoming Wang, Yonghua Yang

**Affiliations:** a State Key Laboratory of Pharmaceutical Biotechnology, School of Life Science, Nanjing University, 22 Hankou Road, Nanjing, 210093, People’s Republic of China

## Abstract

In the crystal structure of the title compound, C_21_H_25_NO_11_, the hexopyranosyl ring adopts a chair conformation and the five substituents are in equatorial positions. An intra­molecular hydrogen bond between the amino group and a neighbouring carbonyl group is found. Two carbonyl groups are disordered and were refined using a split model.

## Related literature

The title compound was first obtained by Robert & Tabone (1953 ▶). For the glycosyl­ation reaction of *N*-hydroxy­phthalimide, see: Cao *et al.* (1995 ▶); Saulius *et al.* (2005 ▶). For the Hofmann rearrangement, see: Aspinall (1941 ▶); Yu *et al.* (2001 ▶).
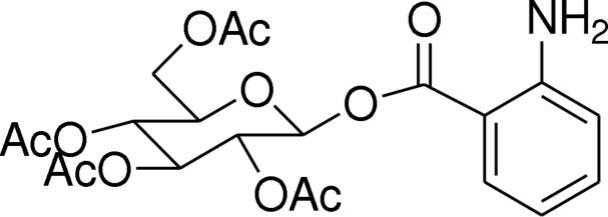


## Experimental

### 

#### Crystal data

C_21_H_25_NO_11_
*M* *_r_* = 467.42
Triclinic, 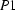

*a* = 5.8220 (12) Å
*b* = 9.1210 (18) Å
*c* = 11.131 (2) Å
α = 98.94 (3)°
β = 94.53 (3)°
γ = 90.22 (3)°
*V* = 582.0 (2) Å^3^
*Z* = 1
Mo *K*α radiation
μ = 0.11 mm^−1^
*T* = 293 K
0.30 × 0.20 × 0.10 mm

#### Data collection

Enraf–Nonius CAD-4 diffractometer
Absorption correction: none
2321 measured reflections
2096 independent reflections
1783 reflections with *I* > 2σ(*I*)
*R*_int_ = 0.020
3 standard reflections every 200 reflections intensity decay: 1%

#### Refinement

*R*[*F* ^2^ > 2σ(*F* ^2^)] = 0.040
*wR*(*F* ^2^) = 0.100
*S* = 1.04
2096 reflections
320 parameters
5 restraints
H-atom parameters constrained
Δρ_max_ = 0.14 e Å^−3^
Δρ_min_ = −0.13 e Å^−3^

### 

Data collection: *CAD-4 EXPRESS* (Enraf–Nonius, 1994 ▶); cell refinement: *CAD-4 EXPRESS*; data reduction: *XCAD4* (Harms & Wocadlo, 1995 ▶); program(s) used to solve structure: *SHELXS97* (Sheldrick, 2008 ▶); program(s) used to refine structure: *SHELXL97* (Sheldrick, 2008 ▶); molecular graphics: *SHELXTL* (Sheldrick, 2008 ▶); software used to prepare material for publication: *SHELXTL*.

## Supplementary Material

Crystal structure: contains datablocks global, I. DOI: 10.1107/S1600536809040975/nc2157sup1.cif

Structure factors: contains datablocks I. DOI: 10.1107/S1600536809040975/nc2157Isup2.hkl

Additional supplementary materials:  crystallographic information; 3D view; checkCIF report

## Figures and Tables

**Table 1:** Hydrogen-bond geometry (Å, °)

*D*—H⋯*A*	*D*—H	H⋯*A*	*D*⋯*A*	*D*—H⋯*A*
N1—H1*B*⋯O11	0.86	2.06	2.704 (5)	131

**Fig. 1.**
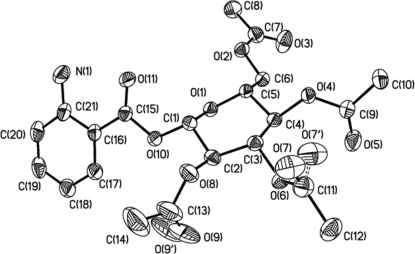
The asymmetric unit of the title compound, showing the atom-labelling scheme. Displacement ellipsoids are drawn at the 50% probability level. The bonds to the disordered atoms are shown with dashed lines. H atoms are omitted for clarity.

## supplementary crystallographic information

### Comment

The title compound was obtained as a by-product of the glycosylation reaction of *N*-hydroxyphthalimide during the synthesis of *O*-(2,3,4,6-Tetra-*O*-acetyl-*β*-*D*-glucopyranosyl)- *N*-oxyphthalimide (Cao *et al.*, 1995; Saulius *et al.*, 2005). Hofmann rearrangement is considered to be the reaction mechanism for its formation (Aspinall,1941;Yu *et al.*,2001).

The hexopyranosyl ring adopts a chair configuration and all substitutents are in equatorial positions (Fig. 1). Between the amino H atoms and the neighboured carbonyl oxygen atom intramolecular N-H···O hydrogen bonding is found (Table 1).

### Experimental

A mixture of *N*-hydroxyphthalimide(1.5 g, 9.2 mmol), tetrabutylammonium hydrogen sulfate (TBAHS 0.34 g, 1 mmol) and Na_2_CO_3_ (1*M*, 20 mL) was stirred at room temperature. After one hour a chloroform solution of 2,3,4,6- tetra-*O*-acetyl-*α*-*D*- glucopyranosyl bromide (3.7 g, 9.0 mmol) was added and the mixture was stirred over night. The organic phase was separated, dried over magnesium sulfate, filtered and concentrated under reduced pressure. Afterwards the product was purified by column chromatography on silica gel(ethylacetate: petroleum ether *v*/*v* 1:2). Removal of the solvent leads to the title compound. Yield: 0.34 g (8%). Single crystals suitable for X-ray analysis were obtained by recrystallization from EtOAc. m.p. 403–405 K.

### Refinement

The H atoms were positioned with idealized geometry (methyl H atoms allowed to rotate but not to tip) and were refined using a riding model. One of the three methyl groups is disordered in two orientations and was refined as disordered group. Two carbonyl groups are disordered and were refined using a split model. Because the absolute structure cannot be determined Friedel opposites were merged in the refinement.

### Crystal data

**Table:**

C_21_H_25_NO_11_	*Z* = 1
*M**_r_* = 467.42	*F*(000) = 246
Triclinic, *P*1	*D*_x_ = 1.334 Mg m^−^^3^
Hall symbol: P 1	Mo *K*α radiation, λ = 0.71073 Å
*a* = 5.8220 (12) Å	Cell parameters from 25 reflections
*b* = 9.1210 (18) Å	θ = 9–12°
*c* = 11.131 (2) Å	µ = 0.11 mm^−^^1^
α = 98.94 (3)°	*T* = 293 K
β = 94.53 (3)°	Block, colourless
γ = 90.22 (3)°	0.30 × 0.20 × 0.10 mm
*V* = 582.0 (2) Å^3^	

### Data collection

**Table:**

Enraf–Nonius CAD-4 diffractometer	*R*_int_ = 0.020
Radiation source: fine-focus sealed tube	θ_max_ = 25.3°, θ_min_ = 1.9°
graphite	*h* = 0→6
ω/2θ scans	*k* = −10→10
2321 measured reflections	*l* = −13→13
2096 independent reflections	3 standard reflections every 200 reflections
1783 reflections with *I* > 2σ(*I*)	intensity decay: 1%

### Refinement

**Table:**

Refinement on *F*^2^	Secondary atom site location: difference Fourier map
Least-squares matrix: full	Hydrogen site location: inferred from neighbouring sites
*R*[*F*^2^ > 2σ(*F*^2^)] = 0.040	H-atom parameters constrained
*wR*(*F*^2^) = 0.100	*w* = 1/[σ^2^(*F*_o_^2^) + (0.0551*P*)^2^ + 0.0615*P*] where *P* = (*F*_o_^2^ + 2*F*_c_^2^)/3
*S* = 1.04	(Δ/σ)_max_ < 0.001
2096 reflections	Δρ_min_ = −0.13 e Å^−^^3^
320 parameters	Extinction correction: *SHELXL97* (Sheldrick, 2008), Fc^*^=kFc[1+0.001xFc^2^λ^3^/sin(2θ)]^-1/4^
5 restraints	Extinction coefficient: 0.052 (8)
Primary atom site location: structure-invariant direct methods	

### Special details

**Table:**

Geometry. All e.s.d.'s (except the e.s.d. in the dihedral angle between two l.s. planes) are estimated using the full covariance matrix. The cell e.s.d.'s are taken into account individually in the estimation of e.s.d.'s in distances, angles and torsion angles; correlations between e.s.d.'s in cell parameters are only used when they are defined by crystal symmetry. An approximate (isotropic) treatment of cell e.s.d.'s is used for estimating e.s.d.'s involving l.s. planes.
Refinement. Refinement of *F*^2^ against ALL reflections. The weighted *R*-factor *wR* and goodness of fit *S* are based on *F*^2^, conventional *R*-factors *R* are based on *F*, with *F* set to zero for negative *F*^2^. The threshold expression of *F*^2^ > σ(*F*^2^) is used only for calculating *R*-factors(gt) *etc*. and is not relevant to the choice of reflections for refinement. *R*-factors based on *F*^2^ are statistically about twice as large as those based on *F*, and *R*- factors based on ALL data will be even larger.

### Fractional atomic coordinates and isotropic or equivalent isotropic displacement parameters (Å^2^)

**Table:**

	*x*	*y*	*z*	*U*_iso_*/*U*_eq_	Occ. (<1)
O1	0.4723 (4)	0.2186 (3)	0.0201 (2)	0.0473 (6)	
O2	0.4296 (4)	0.0007 (3)	−0.1971 (2)	0.0552 (6)	
O3	0.6518 (6)	−0.1311 (4)	−0.3260 (4)	0.1014 (13)	
O4	0.6863 (4)	0.4159 (3)	−0.2092 (2)	0.0478 (6)	
O5	1.0705 (5)	0.4424 (4)	−0.1993 (3)	0.0797 (9)	
O6	0.8323 (4)	0.6042 (3)	0.0311 (2)	0.0539 (6)	
O7	0.6443 (18)	0.8007 (11)	−0.0182 (10)	0.089 (3)	0.50
O7'	0.697 (2)	0.7638 (13)	−0.0826 (9)	0.102 (4)	0.50
O8	0.4552 (5)	0.5912 (3)	0.1864 (2)	0.0642 (7)	
O9	0.715 (3)	0.587 (3)	0.337 (2)	0.172 (11)	0.50
O9'	0.650 (4)	0.562 (2)	0.3641 (18)	0.138 (8)	0.50
O10	0.3348 (4)	0.2984 (3)	0.2025 (2)	0.0538 (6)	
O11	−0.0099 (4)	0.2080 (3)	0.1202 (2)	0.0613 (7)	
N1	−0.2584 (6)	0.0580 (5)	0.2582 (4)	0.0830 (11)	
H1A	−0.3743	0.0071	0.2734	0.100*	
H1B	−0.2509	0.0817	0.1868	0.100*	
C1	0.3810 (6)	0.3425 (4)	0.0908 (3)	0.0468 (8)	
H1C	0.2406	0.3758	0.0493	0.056*	
C2	0.5594 (6)	0.4647 (4)	0.1187 (3)	0.0498 (8)	
H2A	0.6935	0.4331	0.1665	0.060*	
C3	0.6312 (5)	0.5090 (4)	0.0013 (3)	0.0447 (8)	
H3A	0.5061	0.5628	−0.0360	0.054*	
C4	0.6931 (5)	0.3744 (4)	−0.0887 (3)	0.0442 (8)	
H4A	0.8474	0.3406	−0.0653	0.053*	
C5	0.5175 (6)	0.2470 (4)	−0.0987 (3)	0.0423 (7)	
H5A	0.3740	0.2725	−0.1423	0.051*	
C6	0.6126 (6)	0.1085 (4)	−0.1660 (3)	0.0498 (8)	
H6A	0.6767	0.1297	−0.2395	0.060*	
H6B	0.7344	0.0707	−0.1152	0.060*	
C7	0.4769 (8)	−0.1189 (5)	−0.2748 (4)	0.0656 (11)	
C8	0.2885 (9)	−0.2322 (5)	−0.2932 (5)	0.0827 (14)	
H8A	0.2755	−0.2799	−0.3767	0.124*	
H8B	0.1459	−0.1852	−0.2739	0.124*	
H8C	0.3223	−0.3049	−0.2407	0.124*	
C9	0.8873 (6)	0.4456 (4)	−0.2551 (3)	0.0527 (9)	
C10	0.8433 (8)	0.4856 (5)	−0.3784 (4)	0.0667 (11)	
H10A	0.7942	0.5867	−0.3718	0.100*	
H10B	0.7251	0.4214	−0.4236	0.100*	
H10C	0.9822	0.4748	−0.4199	0.100*	
C11	0.8326 (8)	0.7376 (4)	−0.0001 (4)	0.0644 (10)	
C12	1.0535 (8)	0.8176 (5)	0.0358 (5)	0.0797 (13)	
H12A	1.0286	0.9226	0.0444	0.120*	0.50
H12B	1.1587	0.7897	−0.0256	0.120*	0.50
H12C	1.1172	0.7928	0.1122	0.120*	0.50
H12D	1.1744	0.7475	0.0429	0.120*	0.50
H12E	1.0443	0.8804	0.1129	0.120*	0.50
H12F	1.0858	0.8773	−0.0249	0.120*	0.50
C13	0.5261 (15)	0.6275 (7)	0.3044 (5)	0.104 (2)	
C14	0.3672 (15)	0.7450 (8)	0.3642 (6)	0.140 (3)	
H14A	0.4208	0.7742	0.4483	0.210*	
H14B	0.2135	0.7048	0.3589	0.210*	
H14C	0.3675	0.8299	0.3229	0.210*	
C15	0.1294 (6)	0.2281 (4)	0.2076 (3)	0.0463 (8)	
C16	0.1078 (6)	0.1823 (4)	0.3258 (3)	0.0509 (8)	
C17	0.2779 (7)	0.2219 (5)	0.4221 (3)	0.0662 (11)	
H17A	0.4059	0.2771	0.4093	0.079*	
C18	0.2601 (8)	0.1814 (6)	0.5343 (4)	0.0797 (13)	
H18A	0.3756	0.2077	0.5967	0.096*	
C19	0.0679 (8)	0.1007 (6)	0.5540 (4)	0.0797 (13)	
H19A	0.0542	0.0727	0.6300	0.096*	
C20	−0.0983 (8)	0.0629 (5)	0.4637 (4)	0.0734 (12)	
H20A	−0.2259	0.0093	0.4792	0.088*	
C21	−0.0876 (6)	0.1010 (5)	0.3469 (4)	0.0598 (10)	

### Atomic displacement parameters (Å^2^)

**Table:**

	*U*^11^	*U*^22^	*U*^33^	*U*^12^	*U*^13^	*U*^23^
O1	0.0441 (13)	0.0510 (13)	0.0484 (14)	−0.0033 (10)	0.0031 (10)	0.0132 (11)
O2	0.0537 (14)	0.0508 (14)	0.0595 (15)	−0.0097 (11)	0.0062 (11)	0.0030 (12)
O3	0.103 (3)	0.079 (2)	0.117 (3)	−0.0201 (19)	0.056 (2)	−0.026 (2)
O4	0.0410 (12)	0.0614 (14)	0.0442 (13)	−0.0013 (10)	0.0038 (10)	0.0185 (10)
O5	0.0482 (16)	0.115 (3)	0.085 (2)	0.0004 (16)	0.0113 (14)	0.0408 (19)
O6	0.0488 (13)	0.0473 (13)	0.0660 (15)	−0.0081 (10)	−0.0006 (11)	0.0135 (11)
O7	0.097 (6)	0.059 (5)	0.111 (8)	−0.001 (4)	−0.026 (6)	0.027 (6)
O7'	0.137 (9)	0.074 (7)	0.097 (7)	−0.030 (6)	−0.043 (7)	0.048 (6)
O8	0.0817 (19)	0.0653 (17)	0.0434 (14)	−0.0074 (14)	0.0076 (13)	0.0007 (12)
O9	0.195 (14)	0.25 (2)	0.054 (10)	−0.041 (14)	−0.044 (11)	−0.003 (10)
O9'	0.24 (2)	0.120 (9)	0.045 (7)	0.027 (12)	−0.037 (9)	0.012 (6)
O10	0.0464 (13)	0.0762 (17)	0.0416 (13)	−0.0096 (12)	0.0003 (10)	0.0195 (12)
O11	0.0465 (13)	0.0835 (18)	0.0562 (15)	−0.0085 (12)	−0.0038 (12)	0.0225 (13)
N1	0.056 (2)	0.113 (3)	0.084 (3)	−0.024 (2)	0.0081 (19)	0.029 (2)
C1	0.0431 (18)	0.057 (2)	0.0412 (18)	−0.0010 (16)	0.0010 (14)	0.0115 (16)
C2	0.053 (2)	0.055 (2)	0.0421 (17)	−0.0015 (16)	−0.0016 (15)	0.0115 (16)
C3	0.0415 (18)	0.0468 (19)	0.0463 (19)	−0.0008 (15)	−0.0011 (14)	0.0115 (15)
C4	0.0395 (17)	0.051 (2)	0.0440 (18)	−0.0010 (15)	−0.0026 (14)	0.0153 (15)
C5	0.0365 (16)	0.0510 (19)	0.0408 (17)	−0.0019 (14)	0.0005 (13)	0.0127 (14)
C6	0.0476 (19)	0.052 (2)	0.0501 (19)	−0.0045 (16)	0.0055 (15)	0.0068 (16)
C7	0.083 (3)	0.055 (2)	0.057 (2)	−0.010 (2)	0.007 (2)	0.0042 (19)
C8	0.099 (4)	0.064 (3)	0.080 (3)	−0.028 (3)	0.008 (3)	−0.005 (2)
C9	0.050 (2)	0.052 (2)	0.059 (2)	−0.0008 (16)	0.0101 (18)	0.0132 (17)
C10	0.074 (3)	0.074 (3)	0.056 (2)	−0.011 (2)	0.014 (2)	0.019 (2)
C11	0.076 (3)	0.050 (2)	0.069 (3)	−0.007 (2)	0.001 (2)	0.0158 (19)
C12	0.081 (3)	0.059 (3)	0.099 (4)	−0.020 (2)	0.016 (3)	0.007 (2)
C13	0.152 (6)	0.099 (4)	0.052 (3)	−0.029 (4)	−0.004 (4)	−0.006 (3)
C14	0.209 (8)	0.121 (5)	0.081 (4)	−0.021 (5)	0.050 (5)	−0.032 (4)
C15	0.0416 (18)	0.049 (2)	0.0492 (19)	0.0038 (15)	0.0062 (15)	0.0104 (15)
C16	0.047 (2)	0.059 (2)	0.050 (2)	0.0088 (16)	0.0126 (16)	0.0149 (17)
C17	0.061 (2)	0.093 (3)	0.048 (2)	−0.001 (2)	0.0040 (18)	0.021 (2)
C18	0.069 (3)	0.121 (4)	0.053 (2)	0.005 (3)	0.006 (2)	0.028 (2)
C19	0.080 (3)	0.112 (4)	0.060 (3)	0.023 (3)	0.026 (2)	0.041 (2)
C20	0.067 (3)	0.086 (3)	0.078 (3)	0.004 (2)	0.031 (2)	0.034 (2)
C21	0.045 (2)	0.070 (2)	0.069 (2)	0.0069 (18)	0.0152 (18)	0.021 (2)

### Geometric parameters (Å, °)

**Table:**

O1—C1	1.405 (4)	C6—H6A	0.9700
O1—C5	1.430 (4)	C6—H6B	0.9700
O2—C7	1.326 (5)	C7—C8	1.485 (6)
O2—C6	1.432 (4)	C8—H8A	0.9600
O3—C7	1.203 (5)	C8—H8B	0.9600
O4—C9	1.356 (4)	C8—H8C	0.9600
O4—C4	1.447 (4)	C9—C10	1.478 (5)
O5—C9	1.194 (4)	C10—H10A	0.9600
O6—C11	1.316 (4)	C10—H10B	0.9600
O6—C3	1.441 (4)	C10—H10C	0.9600
O7—C11	1.257 (10)	C11—C12	1.472 (6)
O7'—C11	1.217 (9)	C12—H12A	0.9600
O8—C13	1.335 (6)	C12—H12B	0.9600
O8—C2	1.440 (5)	C12—H12C	0.9600
O9—C13	1.204 (17)	C12—H12D	0.9600
O9'—C13	1.169 (14)	C12—H12E	0.9600
O10—C15	1.364 (4)	C12—H12F	0.9600
O10—C1	1.409 (4)	C13—C14	1.526 (11)
O11—C15	1.206 (4)	C14—H14A	0.9600
N1—C21	1.357 (5)	C14—H14B	0.9600
N1—H1A	0.8600	C14—H14C	0.9600
N1—H1B	0.8600	C15—C16	1.456 (5)
C1—C2	1.500 (5)	C16—C17	1.404 (5)
C1—H1C	0.9800	C16—C21	1.410 (5)
C2—C3	1.515 (5)	C17—C18	1.368 (6)
C2—H2A	0.9800	C17—H17A	0.9300
C3—C4	1.522 (5)	C18—C19	1.388 (7)
C3—H3A	0.9800	C18—H18A	0.9300
C4—C5	1.530 (4)	C19—C20	1.342 (7)
C4—H4A	0.9800	C19—H19A	0.9300
C5—C6	1.497 (5)	C20—C21	1.403 (6)
C5—H5A	0.9800	C20—H20A	0.9300
			
C1—O1—C5	112.2 (2)	H10B—C10—H10C	109.5
C7—O2—C6	115.5 (3)	O7'—C11—O7	39.0 (6)
C9—O4—C4	118.9 (3)	O7'—C11—O6	119.0 (6)
C11—O6—C3	120.2 (3)	O7—C11—O6	119.4 (6)
C13—O8—C2	117.4 (4)	O7'—C11—C12	124.0 (7)
C15—O10—C1	117.7 (3)	O7—C11—C12	123.8 (6)
C21—N1—H1A	120.0	O6—C11—C12	112.6 (4)
C21—N1—H1B	120.0	C11—C12—H12A	109.5
H1A—N1—H1B	120.0	C11—C12—H12B	109.5
O1—C1—O10	106.7 (3)	H12A—C12—H12B	109.5
O1—C1—C2	109.6 (3)	C11—C12—H12C	109.5
O10—C1—C2	107.4 (3)	H12A—C12—H12C	109.5
O1—C1—H1C	111.0	H12B—C12—H12C	109.5
O10—C1—H1C	111.0	C11—C12—H12D	109.5
C2—C1—H1C	111.0	H12A—C12—H12D	141.1
O8—C2—C1	107.6 (3)	H12B—C12—H12D	56.3
O8—C2—C3	108.0 (3)	H12C—C12—H12D	56.3
C1—C2—C3	110.1 (3)	C11—C12—H12E	109.5
O8—C2—H2A	110.3	H12A—C12—H12E	56.3
C1—C2—H2A	110.3	H12B—C12—H12E	141.1
C3—C2—H2A	110.3	H12C—C12—H12E	56.3
O6—C3—C2	107.8 (3)	H12D—C12—H12E	109.5
O6—C3—C4	108.3 (3)	C11—C12—H12F	109.5
C2—C3—C4	111.6 (3)	H12A—C12—H12F	56.3
O6—C3—H3A	109.7	H12B—C12—H12F	56.3
C2—C3—H3A	109.7	H12C—C12—H12F	141.1
C4—C3—H3A	109.7	H12D—C12—H12F	109.5
O4—C4—C3	108.6 (3)	H12E—C12—H12F	109.5
O4—C4—C5	105.5 (2)	O9'—C13—O9	28 (2)
C3—C4—C5	112.4 (3)	O9'—C13—O8	128.1 (11)
O4—C4—H4A	110.1	O9—C13—O8	117.2 (13)
C3—C4—H4A	110.1	O9'—C13—C14	120.6 (12)
C5—C4—H4A	110.1	O9—C13—C14	131.9 (12)
O1—C5—C6	107.8 (3)	O8—C13—C14	109.1 (7)
O1—C5—C4	110.3 (2)	C13—C14—H14A	109.5
C6—C5—C4	109.7 (3)	C13—C14—H14B	109.5
O1—C5—H5A	109.7	H14A—C14—H14B	109.5
C6—C5—H5A	109.7	C13—C14—H14C	109.5
C4—C5—H5A	109.7	H14A—C14—H14C	109.5
O2—C6—C5	108.5 (3)	H14B—C14—H14C	109.5
O2—C6—H6A	110.0	O11—C15—O10	120.9 (3)
C5—C6—H6A	110.0	O11—C15—C16	126.9 (3)
O2—C6—H6B	110.0	O10—C15—C16	112.2 (3)
C5—C6—H6B	110.0	C17—C16—C21	118.7 (3)
H6A—C6—H6B	108.4	C17—C16—C15	120.6 (3)
O3—C7—O2	123.0 (4)	C21—C16—C15	120.7 (3)
O3—C7—C8	124.4 (4)	C18—C17—C16	121.6 (4)
O2—C7—C8	112.5 (4)	C18—C17—H17A	119.2
C7—C8—H8A	109.5	C16—C17—H17A	119.2
C7—C8—H8B	109.5	C17—C18—C19	119.3 (4)
H8A—C8—H8B	109.5	C17—C18—H18A	120.4
C7—C8—H8C	109.5	C19—C18—H18A	120.4
H8A—C8—H8C	109.5	C20—C19—C18	120.2 (4)
H8B—C8—H8C	109.5	C20—C19—H19A	119.9
O5—C9—O4	122.9 (3)	C18—C19—H19A	119.9
O5—C9—C10	126.6 (4)	C19—C20—C21	122.7 (4)
O4—C9—C10	110.5 (3)	C19—C20—H20A	118.6
C9—C10—H10A	109.5	C21—C20—H20A	118.6
C9—C10—H10B	109.5	N1—C21—C20	120.4 (4)
H10A—C10—H10B	109.5	N1—C21—C16	122.1 (4)
C9—C10—H10C	109.5	C20—C21—C16	117.5 (4)
H10A—C10—H10C	109.5		
			
C5—O1—C1—O10	177.4 (2)	O1—C5—C6—O2	72.2 (3)
C5—O1—C1—C2	−66.6 (3)	C4—C5—C6—O2	−167.7 (3)
C15—O10—C1—O1	−84.7 (3)	C6—O2—C7—O3	−7.3 (6)
C15—O10—C1—C2	157.9 (3)	C6—O2—C7—C8	174.2 (4)
C13—O8—C2—C1	109.3 (4)	C4—O4—C9—O5	−1.8 (5)
C13—O8—C2—C3	−131.8 (4)	C4—O4—C9—C10	−180.0 (3)
O1—C1—C2—O8	177.5 (3)	C3—O6—C11—O7'	−21.7 (9)
O10—C1—C2—O8	−67.0 (3)	C3—O6—C11—O7	23.3 (8)
O1—C1—C2—C3	60.0 (3)	C3—O6—C11—C12	−179.0 (3)
O10—C1—C2—C3	175.5 (3)	C2—O8—C13—O9'	−7.0 (19)
C11—O6—C3—C2	−124.0 (3)	C2—O8—C13—O9	23.4 (19)
C11—O6—C3—C4	115.1 (4)	C2—O8—C13—C14	−170.0 (4)
O8—C2—C3—O6	73.9 (3)	C1—O10—C15—O11	−1.9 (5)
C1—C2—C3—O6	−168.8 (3)	C1—O10—C15—C16	177.2 (3)
O8—C2—C3—C4	−167.2 (3)	O11—C15—C16—C17	−175.7 (4)
C1—C2—C3—C4	−49.9 (4)	O10—C15—C16—C17	5.2 (5)
C9—O4—C4—C3	101.4 (3)	O11—C15—C16—C21	2.4 (6)
C9—O4—C4—C5	−137.9 (3)	O10—C15—C16—C21	−176.7 (3)
O6—C3—C4—O4	−79.8 (3)	C21—C16—C17—C18	1.2 (6)
C2—C3—C4—O4	161.6 (2)	C15—C16—C17—C18	179.3 (4)
O6—C3—C4—C5	163.8 (2)	C16—C17—C18—C19	−0.8 (7)
C2—C3—C4—C5	45.2 (3)	C17—C18—C19—C20	0.0 (8)
C1—O1—C5—C6	−179.8 (3)	C18—C19—C20—C21	0.3 (8)
C1—O1—C5—C4	60.5 (3)	C19—C20—C21—N1	178.9 (4)
O4—C4—C5—O1	−167.3 (2)	C19—C20—C21—C16	0.1 (7)
C3—C4—C5—O1	−49.1 (3)	C17—C16—C21—N1	−179.6 (4)
O4—C4—C5—C6	74.1 (3)	C15—C16—C21—N1	2.2 (6)
C3—C4—C5—C6	−167.7 (3)	C17—C16—C21—C20	−0.8 (5)
C7—O2—C6—C5	169.0 (3)	C15—C16—C21—C20	−178.9 (4)

### Hydrogen-bond geometry (Å, °)

**Table:**

*D*—H···*A*	*D*—H	H···*A*	*D*···*A*	*D*—H···*A*
N1—H1B···O11	0.86	2.06	2.704 (5)	131
